# Thermodynamics in Material Science

**DOI:** 10.3390/e20070532

**Published:** 2018-07-16

**Authors:** Leslie Glasser, H. Donald Brooke Jenkins

**Affiliations:** 1 Nanochemistry Research Institute, Department of Chemistry, Curtin University, Perth, WA 6845, Australia; 2 Department of Chemistry, University of Warwick, Coventry CV4 7AL, UK

**Keywords:** friction, viscosity, fatigue, thermal effects, metamaterials, nanomaterials, glasses

Authors were invited to offer papers dealing with Thermodynamics in Material Science, noting that thermodynamics has two traditional aspects: the generation of experimental data (then collected into databases) and the theoretical relationships which exist between different data types. 

The papers included in this Special Issue showcase both the strength and the scope of modern-day thermodynamics in the material sciences and related areas, with contributions from Estonia, Russia, China, Poland, Germany, Hungary, USA and Canada. Thus we have presentations dealing with theoretical matters, experimental studies of alloy systems, chemical processes, and the consequences of mechanical processes. In fact, the highly specific nature of many of the papers displays the versatility of modern thermodynamics in its broad coverage of these topics.

This issue considers a number of highly relevant concerns which include:

- A study of the use of Boltzmann and Stirling approaches in theoretical nano-thermodynamic calculations [1] where the replacement of Stirling’s Approximation by the de Moivre approximation is shown to improve results.
- A study of Δ*Η*_mixing_ of Al-Tb alloys in the liquid phase [2]
- A new procedure [3] is proposed for enhancing the performance of a microcooler in both homogeneous and hybrid systems.
- Composite Cu–Cr systems are examined during electric arc exposure [4].
- Metallic liquids form moderately strong glasses [5]. There are clear thermodynamic trends demonstrating, for example, that their kinetic fragility is directly connected to their excess specific heats. This seems to be related to the fact that increasing numbers of differently-sized species in the mix produce greater densities.
- The entropy increase brought about by fatigue occurring in soldering materials [6] is examined and confirmed using experimental studies.
- The behavior of transformation optics in order to monitor heat flux in a new class of metamaterials [7] is reported.
- A comparative study [8] is made of deposition equilibrium of carbon nanotubes compared to graphite for the reforming processes involving liquid petroleum gas (dry process) and natural gas (wet process).
- Studies are related to the modelling of a continuous, microwave-assisted ethyl acetate production process, the aim being to enhance production [9]. The study involves variable initial flow rate velocities coupled with simulation of various temperature profiles which are checked experimentally using optical fibers. The results are relevant to a large scale microwave-induced production process for the ester.
- The frictional properties of diamond films [10] have been studied. Such films are important due to their ability to self-lubricate by the formation of a carbonaceous interface between themselves and the component in contact with them. In the specific study considered the latter components are Si_3_N_4_ balls and, using a non-equilibrium thermodynamic model, various wear mechanisms are modelled. Such studies have relevance for railway rolling stock and other frictionally-dependent areas of interest.

Each of these studies exemplifies the usage of modern thermodynamics in practical applications of some importance in the varying fields of study here embraced.
